# Predicting Volume of Distribution in Neonates: Performance of Physiologically Based Pharmacokinetic Modelling

**DOI:** 10.3390/pharmaceutics15092348

**Published:** 2023-09-19

**Authors:** Pieter-Jan De Sutter, Phebe Rossignol, Lien Breëns, Elke Gasthuys, An Vermeulen

**Affiliations:** Department of Bioanalysis, Ghent University, 9000 Ghent, Belgium

**Keywords:** physiologically based pharmacokinetics (PBPK), pharmacokinetics, neonates, volume of distribution, allometric scaling, developmental pharmacology

## Abstract

The volume of distribution at steady state (Vss) in neonates is still often estimated through isometric scaling from adult values, disregarding developmental changes beyond body weight. This study aimed to compare the accuracy of two physiologically based pharmacokinetic (PBPK) Vss prediction methods in neonates (Poulin & Theil with Berezhkovskiy correction (P&T+) and Rodgers & Rowland (R&R)) with isometrical scaling. PBPK models were developed for 24 drugs using in-vitro and in-silico data. Simulations were done in Simcyp (V22) using predefined populations. Clinical data from 86 studies in neonates (including preterms) were used for comparison, and accuracy was assessed using (absolute) average fold errors ((A)AFEs). Isometric scaling resulted in underestimated Vss values in neonates (AFE: 0.61), and both PBPK methods reduced the magnitude of underprediction (AFE: 0.82–0.83). The P&T+ method demonstrated superior overall accuracy compared to isometric scaling (AAFE of 1.68 and 1.77, respectively), while the R&R method exhibited lower overall accuracy (AAFE: 2.03). Drug characteristics (LogP and ionization type) and inclusion of preterm neonates did not significantly impact the magnitude of error associated with isometric scaling or PBPK modeling. These results highlight both the limitations and the applicability of PBPK methods for the prediction of Vss in the absence of clinical data.

## 1. Introduction

The volume of distribution at steady state (Vss) is a pharmacokinetic (PK) parameter that plays a crucial role in determining the loading dose of a drug and, along with clearance, influences its half-life [1]. The Vss represents the relationship between the amount of drug in the body relative to the plasma concentration at distribution equilibrium. High Vss drugs (e.g., propofol, Vss~4.7 L/kg) have a propensity to enter and/or bind to tissues, while low Vss drugs (e.g., cefazolin, Vss~0.12 L/kg) tend to mostly remain in the bloodstream and/or bind to plasma proteins [2].

Given the ethical and logistical challenges associated with conducting clinical studies in (preterm) neonates, the neonatal PK of many drugs remains unknown [3]. In the absence of clinical data, the neonatal Vss is therefore often estimated by scaling it isometrically from an adult value using body weight as the only influencing parameter (i.e., using the same Vss per kg weight as in adults) [4]. However, neonates differ from adults not only in body weight but also in body composition. For instance, neonates possess a higher extracellular body water content and a lower fat mass proportion than adults [5]. These physiological differences are expected to significantly impact the Vss of drugs, especially for hydrophilic and polar compounds, which preferentially distribute into water. Consequently, the Vss of such compounds in neonates is often higher than expected when scaled from adults [4]. Neonates also exhibit lower concentrations of plasma proteins, such as albumin [6] and alpha_1_-acid glycoprotein [7], leading to a higher free fraction of drugs in plasma [6,8]. This altered protein binding can further contribute to higher Vss values in neonates.

Considering these complexities, the use of physiologically based pharmacokinetic (PBPK) modeling emerges as a promising approach to predict PK in neonates [9]. PBPK modeling integrates drug-specific data and body physiology, providing a comprehensive framework of drug disposition. For the prediction of Vss, PBPK modeling typically employs tissue composition-based models. The Poulin and Theil method, for example, calculates tissue-to-plasma partition coefficients (Kps) as a function of drug lipophilicity, fraction unbound in plasma and the abundance of lipids in tissues [10]. Berezhkovskiy later refined this method by also considering the ratio of the unbound fraction in tissue to that in plasma [11]. The Rodgers and Rowland method further builds upon these methodologies by splitting tissues into intra- and extracellular components, incorporating drug ionization (i.e., acid-base character) and parameterizing phospholipid binding using the blood-to-plasma ratio (BP) [12]. 

These PBPK modeling methods are particularly attractive for predicting neonatal Vss as they can account for the changing tissue composition and plasma protein concentrations. However, these methods were primarily developed to predict Vss in adults, and their accuracy in predicting neonatal Vss remains uncertain. Furthermore, while over the past two decades, PBPK modeling has gained traction in predicting pediatric PK [13], it has primarily been evaluated for the prediction of the clearance of drugs [14,15,16] and its application to the (preterm) neonatal population is still limited [9]. Therefore, the primary objective of this study was to assess the performance of PBPK modeling in predicting neonatal Vss. This was done by comparing Vss predicted by two methods (Poulin & Theil with Berezhkovskiy correction and Rodgers & Rowland) with clinical data extracted from the literature. Secondary objectives were to compare these outcomes with isometric scaling and to identify covariates explaining observed errors.

## 2. Materials and Methods

### 2.1. PBPK Substrate Models

Physiologically based pharmacokinetic (PBPK) substrate models were developed using the Simcyp simulator (V22, Certara, Inc., Princeton, NJ, USA). Drugs from the ATC (Anatomical Therapeutic Chemical code) classes N (Nervous system) and J01C-D (Beta-lactam antibacterial) indicated for use in neonates according to the Dutch Pediatric Formulary [17] were included if a suitable clinical PK study in neonates was available (see below). 

The substrate models were parameterized using in-vitro/in-silico parameters collected from the literature. Molecular weight, LogP and fu were taken from the Lombardo dataset [2]. Ionization type and pKa were sourced from the Williams et al. dataset [18]. Predicted pKa values from the Chemaxon method (as reported in Drugbank online) were used for compounds not present in the Williams et al. dataset. BP ratios were obtained from the Mamada et al. database [19], and for compounds not included in this database, BP ratios were predicted using the internal Simcyp model (bases and neutral compounds) or assumed to be 1 (acids and ampholytes). Two full PBPK models were developed for each drug: one using the Vss prediction method proposed by Poulin & Theil [10] (with Berezhkovskiy correction [11], henceforth called “Poulin & Theil+”) and another using the method proposed by Rodgers & Rowland [12]. These methods correspond to “Method 1” and “Method 2” in the simulator. The third method available in Simcyp (adapted Rodgers & Rowland method) was not evaluated as it is not used by PBPK platforms other than Simcyp. These methods estimate tissue-to-plasma partition coefficients in twelve different tissues: adipose, bone, brain, gut, pancreas, heart, kidney, liver, lung, muscle, skin and spleen. Explicit modelling of these tissues as compartments in the PBPK models allows for mechanistic predictions of Vss. As Vss is independent of clearance, the elimination process was arbitrarily specified as a renal clearance of 1 L/h for each drug. Similarly, as both methods model the Vss as a dose-independent parameter, the administered dose was specified as an intravenous bolus of 1 mg/kg body weight for each drug/simulation.

### 2.2. Clinical Data

Suitable clinical PK studies in neonates were identified through a comprehensive search of the Pubmed database. The search string was: “(“Pharmacokinetic*” OR “PK” OR “distribution”) AND (neonat* OR newborn*) AND <drug name>”. Studies were included if they reported Vss per kg bodyweight after intravenous administration in at least three neonates. For the purpose of this work, neonates are defined as having a postnatal age (PNA) of a maximum of 60 days. If the study also included children older than 60 days, the study was included only if the Vss could be aggregated, excluding the data from non-neonates. Studies involving neonates during extracorporeal membrane oxygenation (ECMO) or cardiopulmonary bypass (CPB) were excluded due to the possibility of alterations in blood volume, which may affect Vss. Additional data extracted from the studies included the number of neonates (N), the proportion of females, minimum and maximum PNA, minimum and maximum gestational age (GA) and proportion of preterm neonates. For the purpose of this work, neonates were defined as preterm if their gestational age was less than 37 weeks. Studies were further categorized into sub-studies consisting of term and/or preterm neonates when possible. Adult reference Vss values per kg bodyweight were obtained from the Lombardo et al. dataset [2].

### 2.3. PBPK Simulations

PBPK simulations were performed using predefined virtual populations in the Simcyp simulator (V22), namely “Sim-Paediatric” for term neonates, “Sim-Preterm” for preterm neonates, and “Sim-Healthy volunteers” for adults. Both theoretical simulations and clinical trial-mimicking simulations were carried out.

Theoretical simulations aimed to assess the behavior of the models at fixed GA and PNA. For these simulations, 50 male and 50 female subjects were simulated with a GA of 30 weeks using the preterm population and a PNA of 7 days using the pediatric population. Adult reference Vss values were predicted using an age range between 20 and 25 years old. 

Trial-mimicking simulations were run by sampling subjects from the virtual populations conditional on the study design and subject characteristics of the included clinical PK studies in neonates. More specifically, the simulations were matched in terms of the number of subjects, the proportion of females, PNA and GA (preterm population only). In cases where the study population consisted of both term and preterm neonates, simulations were performed using both virtual populations, and a weighted average of Vss was calculated. If the proportion of preterm neonates was unclear, an equal size of preterms and terms was assumed. Additionally, when GAs were not reported, the neonates were assumed to be at term. Each virtual trial was repeated ten times, and an average Vss value was taken as output. 

### 2.4. Quantitative Analyses

The Vss predictions from the PBPK simulations were compared with observed clinical data. Allometrically scaled neonatal Vss were also calculated for each study, using Equation (1);

(1) $$V_{ss,pred}=V_{ss,adults}\times \left(\frac{{BW}_{adult}}{{BW}_{neonate}}\right)\times {\left(\frac{{BW}_{neonate}}{{BW}_{adult}}\right)}^{\alpha }$$

where V_ss,pred_ is the predicted Vss in neonates (L/kg) and V_ss,adults_ is the observed Vss in adults (L/kg), as reported in the Lombardo et al. database. Alpha (α) is the allometric exponent, and BW is the body weight (kg). BW_neonate_ is the mean or median BW reported in the study. If no BW was reported (9 cases), the median body weight across studies and grouped by subpopulation was inputted. This was 1.19 kg for preterm neonates, 3.23 kg for term neonates and 2.44 kg for a mixed population. As BW is not reported in the Lombardo et al. database, the adult BW (BW_adult_) was put at 70 kg for each study. The primary exponent evaluated was 1.00, and this approach is henceforth denoted as “isometric scaling”. As the BW terms cancel out for isometric scaling, the assumptions and limitations regarding the use of mean BW do not affect the accuracy of this method. 

For both types of analyses (PBPK and allometry), predicted and observed Vss (*V_ss,pred_* and *V_ss,obs_*) were compared by calculating the fold error (FE) for each study, using Equation (2);

(2) $$FE=\frac{V_{ss,pred}}{V_{ss,obs}}$$

A FE smaller than 1 indicates an underprediction, while a FE larger than 1 denotes an overprediction compared to observed values. The FEs were then aggregated across studies by calculating the average fold error (AFE) and absolute average fold error (AAFE) using Equations (3) and (4), respectively:

(3) $$AFE=10^{\frac{1}{n}\sum \log(FE)}$$

(4) $$AAFE=10^{\frac{1}{n}\sum \left|\log(FE)\right|}$$

where n is the number of studies, and FE is the fold error calculated using equation 2. The AFE indicates the overall directional error (~bias, i.e., under- or overprediction), while the AAFE signifies the overall error, irrespective of direction (~precision). Additionally, the percentage of studies for which the predicted Vss lies within two-fold of the observed (reported) Vss was calculated. 

Covariate analyses on the AFE on Vss were either done using a linear model (continuous covariate: LogP) or by using the Kruskal-Wallis test (categorical covariates: ionization type and neonatal population type). AFE values were log-transformed prior to covariate analysis and back-transformed for interpretation. All quantitative analyses were carried out in R version 4.3.1 [20]. 

## 3. Results

### 3.1. Accuracy of PBPK Predicted Adult Vss 

**Figure 1:**
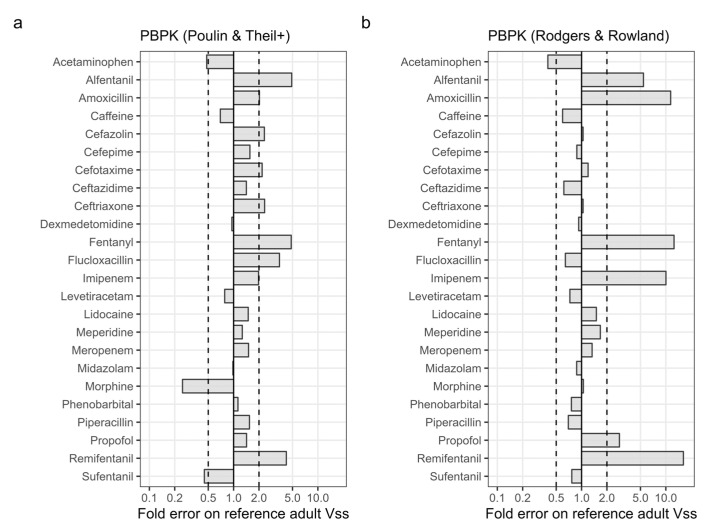
Accuracy of physiologically based pharmacokinetic (PBPK) predictions of the volume of distribution at steady state (Vss) in adults. PBPK predictions are made with either the Poulin & Theil with Berezhkovskiy correction method (panel **a**) or the Rodgers & Rowland method (panel **b**). Fold errors represent the ratio of the PBPK prediction to the reference adult Vss value, as taken from the Lombardo et al. database [2].

PBPK models were developed for 24 drugs, using in-vitro and in-silico parameter values drawn from the scientific literature (Table 1). To evaluate the predictive capability of these models for estimating the Vss in adult individuals, the model predictions for an adult population were compared with observed Vss values obtained from the Lombardo et al. database. The outcomes of this analysis, displayed in Figure 1 for each drug, along with the aggregated accuracy metrics in Table 2, indicate a tendency for both PBPK models to overestimate adult Vss values by an average of ~50% (AFE 1.45–1.54). The Rodgers & Rowland method outperformed the Poulin & Theil+ method for a larger proportion of drugs (71% versus 54% of the drugs with predictions within two-fold). However, when the Rodgers & Rowland method yielded inaccurate predictions, the errors were typically larger than with the Poulin & Theil+ method. This is also reflected by the slightly larger AAFE value associated with the Rodgers & Rowland method (2.14 versus 1.95). 

To test the robustness of these results, the analyses were repeated using alternative observed adult Vss data collected from individual studies instead of the reference data reported in the Lombardo et al. database (Supplementary Table S1). All alternative Vss values were within a two-fold of the reference data, except for fentanyl (overpredicted) and sufentanil (underpredicted) (Supplementary Figure S1a). Overall accuracy was slightly worse for both PBPK methods when alternative adult Vss estimates were used as reference (AAFE of 2.10 and 2.19 for the Poulin & Theil+ and Rodgers & Rowland methods, respectively) (Supplementary Figure S1b,c). In the subsequent analyses, the Lombardo et al. dataset was used as the only source for adult Vss data. 

### 3.2. Variability of PBPK Input Parameters

To characterize the variability associated with the input parameters of the PBPK models, alternative parameter values (Supplementary Table S2) were compared with the original data. Alternative LogP values, sourced from Drugbank, did not differ more than 1 unit from the original input values, except for ceftriaxone and imipenem (Supplementary Figure S2a). Similarly, Chemaxon predicted pKa values did not differ more than one unit compared to experimental values reported in the Williams et al. database, except for flucloxacillin (Figure S2b). Free fractions reported in Drugbank generally concurred with the values sourced from the Lombardo et al. dataset (Supplementary Figure S2c). For the drugs with experimental BP ratios, the predicted (bases and neutral compounds) or assumed (acids) BP ratios were all within two-fold of the experimental data (Supplementary Figure S2d).

For the 14 drugs for which alternative values for LogP, pKa, fu and BP were found, PBPK models were developed using these alternative inputs and predicted adult Vss were compared with the predicted adult Vss using the original PBPK models (Supplementary Figure S3). Using the alternative input parameters had limited influence on the predicted adult Vss estimates when the Poulin & Theil+ method was used (AAFE: 1.30, one drug underpredicted: alfentanil). Larger discrepancies were seen with the Rodgers & Rowland method (AAFE: 1.49, and three drugs had predictions outside the two-fold range: alfentanil, lidocaine and meperidine). All following simulations were done with the original models.

### 3.3. PBPK Predicted Changes in Neonatal Vss

**Figure 2:**
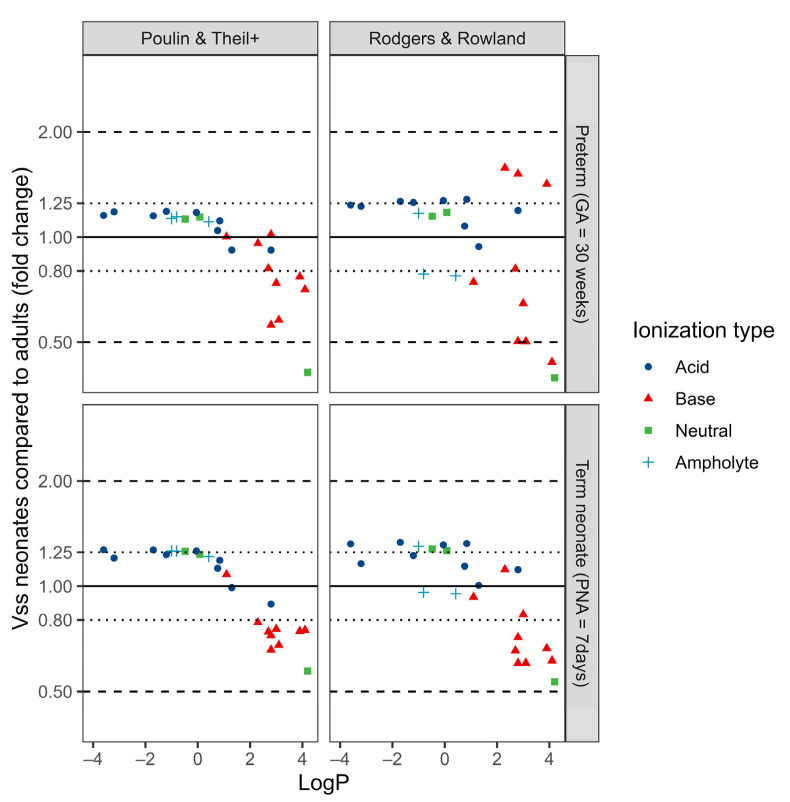
Physiologically based pharmacokinetic (PBPK) predicted neonatal volume of distribution at steady state (Vss) as a function of drug lipophilicity (LogP). The Vss in neonates is indicated as a fold change compared with predicted Vss values in adults, with a fold change of 1 indicating no difference between predictions in neonates and adults. PBPK predictions were made with two methods: Poulin & Theil with Berezhkovskiy correction (panels on the **left**) and the Rodgers & Rowland method (panels on the **right**). Results are presented for preterms at birth with a gestational age (GA) of 30 weeks (**top** panels) and for term neonates with a postnatal age of 7 days (**bottom** panels).

Subsequently, the developed PBPK models were used to predict the Vss in term- and preterm neonates. To facilitate interpretation, the Vss values in neonates were expressed as fold changes relative to the PBPK-predicted Vss values in adults and plotted as a function of drug lipophilicity (LogP) (Figure 2). Generally, the predicted neonatal Vss did not consistently differ more than two-fold from adult values, except for propofol in preterm neonates (FE of 0.38 for both methods). Fentanyl, midazolam and dexmedetomidine had fold errors lower than 0.5 in preterm neonates (0.41, 0.49 and 0.49, respectively), but only when the Rodgers & Rowland method was used.

In term neonates, both models predicted increased Vss for hydrophilic drugs (LogP < 2) and decreased Vss for lipophilic drugs (LogP > 2). For preterm neonates, the Poulin and Theil+ model predicted slightly increased Vss for hydrophilic drugs and decreased Vss for lipophilic drugs. For the Rodgers & Rowland method, however, no noticeable trend can be discerned regarding Vss changes in preterm neonates and lipophilicity, except for a larger variability at higher LogP. The interpretation of these results might be confounded by the ionization type, as most lipophilic drugs included are bases (cationic), while most hydrophilic drugs are acids (anionic).

### 3.4. Accuracy of Predicted Neonatal Vss

A comprehensive literature search was conducted to identify studies reporting the Vss of the selected drugs in neonates. This search yielded 66 publications suitable for inclusion, from which 86 (sub)studies were extracted and summarized in Supplementary Table S3. These studies encompassed 31 investigations in term neonates, 29 in preterm neonates and 26 involving both term and preterm neonates. The overall population size included 3343 neonates, with a median of 14 neonates per study (ranging from 3 to 875). On a per-drug basis, a median of 70 neonates (ranging from 8 to 925) across three studies (ranging from 1 to 10) were included.

**Figure 3:**
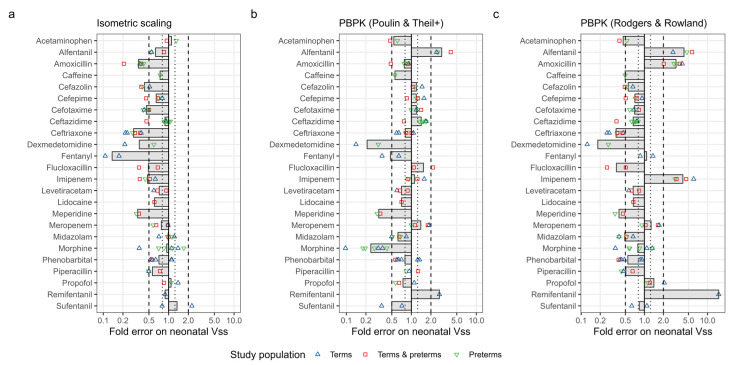
Accuracy of isometric scaling (panel **a**) and physiologically based pharmacokinetic (PBPK) modeling to predict the volume of distribution at steady state (Vss) in neonates (panels **b**,**c**). PBPK predictions are made with either the Poulin & Theil with Berezhkovskiy correction method (panel **b**) or the Rodgers & Rowland method (panel **c**). Fold errors represent the ratio of the PBPK prediction to the observed Vss in neonates. Each symbol represents a separate study, while the grey columns represent average fold errors.

In the next step, the collected observed neonatal Vss values were compared to isometrically scaled Vss values, and PBPK predicted the Vss values using a neonatal population. The results of these analyses are presented in Figure 3, together with aggregated accuracy metrics in Table 2.

#### 3.4.1. Isometric and Allometric Scaling of Neonatal Vss

Isometric scaling generally underestimated neonatal Vss, with an overall AFE of 0.61. Notably, the largest underprediction was observed for fentanyl, with an AFE of 0.14 (Figure 3a). Significant variability between studies was evident for certain drugs. For example, morphine Vss exhibited accurate predictions on average (AFE: 0.94), but the fold errors of individual studies ranged from 0.36 to 1.70, highlighting underlying discrepancies.

**Figure 4:**
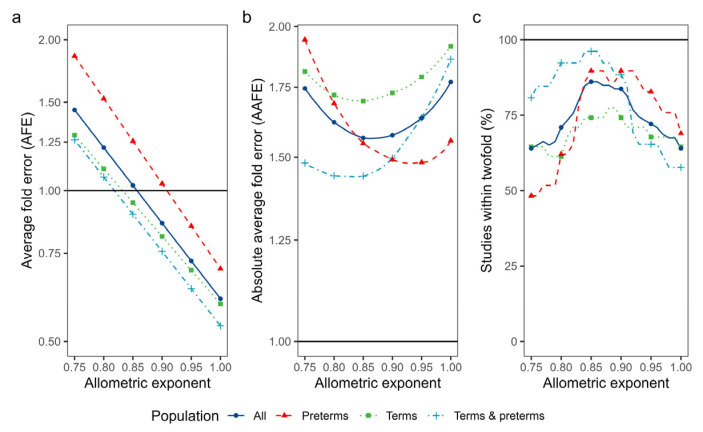
Influence of using different allometric exponents on accuracy metrics. The solid vertical black lines denote the accuracy target: an average fold error (AFE) and absolute average fold error (AAFE) of 1 (panels **a** and **b**, respectively) and 100% of the studies within two-fold of observed values (panel **c**). The allometric exponent, which minimizes the distance between the colored line and the accuracy target, is the optimal exponent. The solid blue lines denote the accuracy of the overall population (i.e., all studies), while the dashed red, dotted green and dot-dashed azure lines represent the accuracy using studies with only preterm neonates, only term neonates or a mixed population of term and preterm neonates, respectively.

As isometric scaling yielded underpredictions, the effect of applying allometric exponents between 0.75 (common allometric exponent for clearance) and 1.00 (isometric scaling) on the accuracy metrics was tested (Figure 4). Using an allometric exponent of 0.85 yielded the most accurate results (AFE: 1.02, AAFE: 1.56, 86% of studies within two-fold). When the results were separated according to term/preterm status, an allometric exponent of 0.90 seemed best for preterm neonates, while for term neonates and mixed populations, 0.85 remained optimal. However, the results regarding allometric scaling should be interpreted with caution as mean body weights per study were used, and an adult body weight of 70 kg was assumed for all cases.

#### 3.4.2. PBPK Modelling of Vss

The Poulin & Theil+ method reduced the trend of underpredictions (AFE: 0.82) and demonstrated greater accuracy than isometric scaling. This was indicated by a smaller AAFE (1.68 versus 1.77) and a higher percentage of studies predicted within two-fold of observed values compared to isometric scaling (76% versus 64%). The largest inaccuracies with this method were observed for dexmedetomidine (AFE: 0.21) and morphine (AFE: 0.24). 

Similarly, the Rodgers & Rowland method also mitigated the trend of underprediction observed with isometric scaling (AFE: 0.83). However, the overall accuracy was inferior to isometric scaling, indicated by a higher AAFE of 2.03 and a lower percentage of studies predicted within two-fold (55%). The largest inaccuracies with this method were noted for remifentanil (AFE: 15.00) and dexmedetomidine (AFE: 0.18).

#### 3.4.3. Covariate Analysis

**Figure 5:**
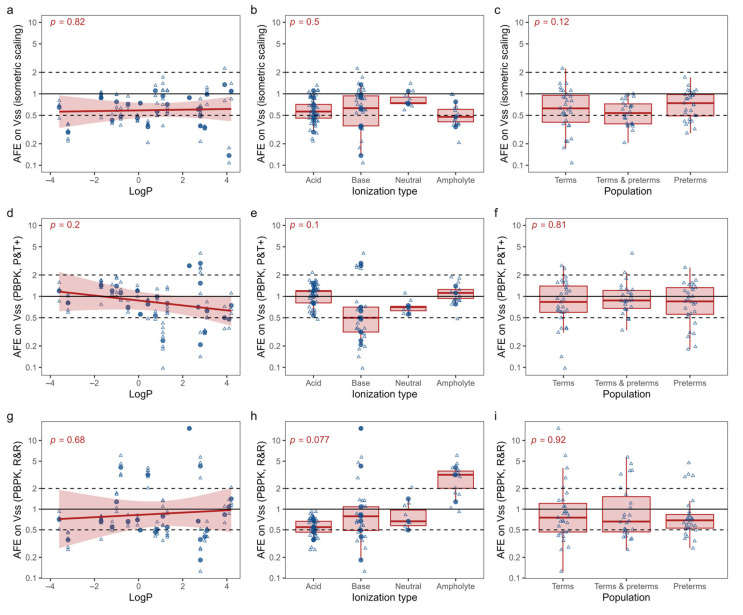
Covariate analysis of the influence of LogP, ionization type and study population (i.e., term- and/or preterm neonates) on the error associated with predicting the volume of distribution at steady state (Vss) using three different methods. The methods evaluated are (i) isometric scaling from adult reference data (panels **a**–**c**), (ii) physiologically based pharmacokinetic modelling (PBPK) using the Poulin & Theil with Berezhkovskiy correction method (P&T+; panels **d**–**f**) and (iii) PBPK modeling using the Rodgers and Rowland method (R&R; panels **g**–**i**). Errors are expressed as fold errors for individual studies (small open triangles) or average fold errors (AFE) on aggregated drug data (larger filled dots). *P* values are calculated using a linear model (panels **a**,**d**,**g**) or by using the Kruskal-Wallis tests (panels **b**,**c**,**e**,**f**,**h**,**i**).

To explore potential factors influencing accuracy variations among drugs and studies using these three methods, subgroup analyses were conducted considering the LogP and ionization type of the drug, as well as the composition of the study population in terms of term and/or preterm neonates. However, the results presented in Figure 5 revealed that neither the hydrophobicity (logP) nor the acid-base character of the drug significantly impacted the errors associated with isometric scaling or PBPK predictions. Additionally, no significant differences were observed in subgroup analyses based on the inclusion of term and/or preterm neonates in the study population.

## 4. Discussion

The use of PBPK modelling has gained considerable traction in predicting PK in children [13]. However, its application in (preterm) neonates is still limited, and its predictive performance, particularly in terms of the Vss, remains largely unexplored. Therefore, this study aimed to evaluate the accuracy of two Vss prediction methods in neonates, utilizing data from 86 clinical studies involving 24 different drugs. Overall, the Poulin & Theil method performed better than isometric scaling, but the Rodgers & Rowland method did not exceed the predictive performance of isometric scaling.

The accuracy of predicted adult Vss (54–71% within two-fold) was similar to assessments made by other authors. For instance, Jones et al. reported that the Poulin & Theil and Rodgers & Rowland methods predicted adult Vss within two-fold for 61% and 50% of 18 drugs, respectively [38]. Similarly, Chan and colleagues evaluated the accuracy of the Rodgers and Rowland method for 152 drugs and found that 66% of drugs were predicted within two-fold [39], consistent with the performance reported by the original authors of the Rodgers and Rowland method (64% within two-fold) [12].

Scaling the adult Vss values isometrically to neonates resulted in underpredicted neonatal Vss for almost all drugs. Using an allometric exponent of 0.85 or 0.90 for term and preterm neonates, respectively, decreased underpredictions and improved overall accuracy. This result is similar to the work of Mahmood, who determined, based on Vss estimates for 16 drugs in low birth weight neonates, that isometrically scaled Vss was associated with prediction errors higher than 50% (i.e., ~AFE < 0.5) in 29% of cases. When an allometric exponent of 0.90 was used, the percentage of Vss predictions with prediction errors higher than 50% decreased to 21% [4]. As our allometric analysis utilized aggregated study data, the results are prone to ecological bias [40] and should be interpreted with caution. Before the proposed allometric exponents can be used to scale adult Vss to neonatal Vss, they should be confirmed using individual patient data, which was unavailable for our analysis.

Interestingly, the drug characteristics, such as lipophilicity and ionization type, did not impact the magnitude of error in isometric scaling. This contradicts the common assumption that hydrophilic drugs would have a larger Vss in neonates due to their lower percentage of adipose tissue and higher water content compared to adults, while lipophilic drugs would have a smaller Vss for the same reason [5,41,42]. This assumption is also apparent in the PBPK model, as indicated by the theoretical simulations. However, it should be noted that the difference between adult and neonatal Vss was generally small, while the variability in observed neonatal Vss between studies was, in many cases, extensive. Consequently, the covariate analyses may have lacked the power to detect a clear relationship between lipophilicity and neonatal Vss. Therefore, solid evidence supporting the influence of lipophilicity on the neonatal ontogeny of Vss is currently lacking.

Considering the added uncertainties regarding the neonatal ontogeny of physiological input parameters in PBPK Vss prediction methods [43,44], it is reasonable to expect that Vss predictions in the neonatal population would be less accurate than those made for adults. Surprisingly, the Rodgers & Rowland method exhibited comparable accuracy in adults and neonates (smaller AAFE, but less studies within two-fold) and the Poulin & Theil+ method performed slightly better in neonates than in adults (smaller AAFE and more studies within two-fold). Similarly, one might expect worse predictive performance in preterm than in term neonates, but this was not apparent. 

Both PBPK Vss prediction methods explicitly model the interaction between the drug and binding components in plasma (e.g., albumin) and tissues (neutral lipids and neutral phospholipids). The Rodgers and Rowland method additionally accounts for pH-dependent partitioning between intra- and extracellular water compartments of tissues and binding to acidic phospholipids. The theoretical benefit of PBPK modelling over isometric scaling is that developmental changes impacting these mechanisms can be accounted for through ontogeny functions (e.g., describing lower albumin levels). Interestingly, only the “simpler” Poulin & Theil+ PBPK prediction method achieved superior accuracy compared to isometric scaling, suggesting that the additional mechanisms included in the Rodgers and Rowland fail to explain the residual differences between the predicted and observed Vss. However, as the Rodgers and Rowland method relies more heavily on the accurate specification of drug-related properties (see Supplementary Figure S3), an explanation for the poorer performance of this method can also be sought in inaccuracies in drug parameters (e.g., assumptions regarding blood-to-plasma ratio).

These results give some general confidence in the specification of the virtual neonatal populations and in the applicability of the Vss prediction methods in neonates. However, the clinical applicability of the models should be evaluated on a case-by-case basis, factoring in drug toxicity and ontogeny of other PK parameters such as clearance.

A limitation of this work is that the virtual PBPK populations represented “healthy” neonates, while the clinical data almost exclusively stemmed from severely ill neonates. Comorbidities or therapeutic interventions such as sepsis [45], mechanical ventilation [46], asphyxia [47], congenital heart disease [48] and therapeutic hypothermia [49] may affect Vss through mechanisms such as acidosis, increased vascular permeability or hemodynamic changes. The inclusion of neonates with these characteristics in the current study might be responsible for the observed between-study variability and might have potentially obscured the developmental aspects of Vss. However, the magnitude and clinical relevance of these comorbidities on Vss remains poorly understood. Further work should focus on understanding disease effects on neonatal PK and look at ways to incorporate these effects into virtual PBPK populations.

Further exploration is also needed to investigate whether the observed changes in neonatal Vss translate to differences in the rate of distribution and relevant tissue/effect-site concentrations. The applied PBPK models assume perfusion-limited distribution, which means that the crossing of epithelial membranes is not modelled as the rate-limiting step [50]. However, this assumption may not hold true for the distribution of hydrophilic drugs to organs with tight endothelial junctions, such as the brain [26]. When permeability impacts drug distribution, perfusion-limited models might accurately estimate the Vss, but the shape of the concentration-time profile might be impacted, as it will likely take longer to reach distribution equilibrium. Additionally, influx and efflux transporters, which are not incorporated in the evaluated methods, might alter the rate and extent of drug distribution to target organs and, in case of distribution to larger organs such as the liver, significantly impact Vss [51]. Moreover, the involvement of transporters in processes affecting both elimination and distribution (e.g., renal uptake) can also violate the model assumption of clearance- and dose-independent distribution at steady state. Currently, knowledge of the barriers to the distribution of drugs to developing organs is limited [52], and it is not feasible to directly measure tissue concentrations in vivo. However, integration of in-vitro data (e.g., transporter ontogeny [53]) and preclinical animal data (e.g., from distribution studies in piglets [54]) might contribute to partially filling this gap.

In conclusion, this study evaluated the predictive performance of Vss estimation methods in neonates using PBPK modeling. The Poulin & Theil method with Berezhkovskiy correction exhibited improved predictive accuracy compared to isometric scaling, while the Rodgers & Rowland method did not surpass the predictive performance of isometric scaling. The influence of drug characteristics, such as lipophilicity, on neonatal Vss remains inconclusive, emphasizing the need for further research.

## Figures and Tables

**Table 1:** Input parameter values for the physiologically based pharmacokinetic (PBPK) models.

Drug	MWT (g/mol) ^a^	LogP ^a^	Ionization Type (pKa)	fu ^a^	Main Binding Protein	BP
Acetaminophen	151	0.76	Monoprotic acid (9.7) [18]	0.520	HSA [21]	1.04 [19]
Alfentanil	417	2.80	Monoprotic base (6.5) [18]	0.086	AGP [22]	0.63 [19]
Amoxicillin	365	0.42	Ampholyte (2.4, 9.6) [18]	0.850	HSA [23]	1.04 [19]
Caffeine	194	0.08	Neutral	0.640	HSA [24]	1.00 [19]
Cefazolin	455	−1.20	Monoprotic acid (2.1) [18]	0.180	HSA [25]	0.60 [19]
Cefepime	481	−3.60	Monoprotic acid (2.8) ^b^	0.780	HSA [25]	0.74 [19]
Cefotaxime	455	−0.05	Monoprotic acid (3.4) [18]	0.600	HSA [25]	1.00 ^c^
Ceftazidime	547	−1.70	Diprotic acid (1.8, 2.7) [18]	0.790	HSA [25]	0.72 [19]
Ceftriaxone	555	−3.20	Diprotic acid (3.2, 4.1) [18]	0.100	HSA [25]	1.00 ^c^
Dexmedetomidine	200	2.80	Monoprotic base (6.5) ^b^	0.060	HSA [26]	0.80 ^d^
Fentanyl	336	4.10	Monoprotic base (8.4) [18]	0.160	HSA [27]	0.99 [19]
Flucloxacillin	454	2.80	Monoprotic acid (2.7) [18]	0.043	HSA [28]	1.00 ^c^
Imipenem	299	−0.81	Ampholyte (3.4, 10.9) ^b^	0.860	HSA [29]	1.00 ^c^
Levetiracetam	170	−0.48	Neutral	0.900	HSA ^e^	0.69 ^d^
Lidocaine	234	2.70	Monoprotic base (7.8) [18]	0.330	AGP [30]	0.78 [19]
Meperidine	247	3.00	Monoprotic base (8.7) [18]	0.420	HSA [31]	1.01 [19]
Meropenem	383	−1.00	Ampholyte (3.3, 9.4) ^b^	0.870	HSA ^e^	0.507 [19]
Midazolam	326	3.10	Monoprotic base (6.2) [18]	0.017	HSA [32]	0.68 [19]
Morphine	285	1.10	Monoprotic base (8.0) [18]	0.650	HSA [33]	1.00 [19]
Phenobarbital	232	1.30	Monoprotic acid (7.4) [18]	0.490	HSA [34]	0.99 [19]
Piperacillin	518	0.84	Monoprotic acid (3.5) ^b^	0.500	HSA ^e^	0.65 [19]
Propofol	178	4.20	Neutral	0.016	HSA [35]	1.25 [19]
Remifentanil	376	2.30	Monoprotic base (7.5) ^b^	0.300	AGP [36]	0.90 ^d^
Sufentanil	387	3.90	Monoprotic base (8.0) [18]	0.075	AGP [37]	0.74 [19]

AGP: alpha_1_-acidic glycoprotein, BP: blood-to-plasma ratio, fu: free fraction in plasma, HSA: human serum albumin, MWT: molecular weight, Vss: volume of distribution at steady state. ^a^: data from Lombardo et al. dataset. ^b^: Predicted by Chemaxon (Drugbank). ^c^: no data, assumed. ^d^: predicted by Simcyp V22 from physicochemical data. ^e^: assumed (AGP for monoprotic bases, albumin for other ionization types).

**Table 2:** Accuracy of methods for the prediction of volume of distribution at steady state (Vss).

	Prediction Method	AFE	AAFE	Studies within Two-Fold (%)
Predicting adult Vss (L/kg):				
	PBPK (Poulin & Theil+)	1.45	1.95	54
	PBPK (Rodgers & Rowland)	1.54	2.14	71
Predicting neonatal Vss (L/kg):				
	Isometric scaling	0.61	1.77	64
	PBPK (Poulin & Theil+)	0.82	1.68	76
	PBPK (Rodgers & Rowland)	0.83	2.03	55

AAFE: absolute average fold error, AFE: average fold error, PBPK: physiologically based pharmacokinetics.

## Data Availability

The data presented in this study are available on request from the corresponding author.

## Supplementary Materials

The following supporting information can be downloaded at https://www.mdpi.com/article/10.3390/pharmaceutics15092348/s1: “Supplementary data”, which contains supplementary Figures S1–S3 and supplementary Tables S1–S3. References [55,56,57,58,59,60,61,62,63,64,65,66,67,68,69,70,71,72,73,74,75,76,77,78,79,80,81,82,83,84,85,86,87,88,89,90,91,92,93,94,95,96,97,98,99,100,101,102,103,104,105,106,107,108,109,110,111,112,113,114,115,116,117,118,119,120,121,122,123,124,125,126,127,128,129,130,131,132,133,134,135,136,137,138,139,140,141,142] are cited in the Supplementary Materials.
