# Supplementary material for: Predicting Volume of Distribution in Neonates: Performance of Physiologically Based Pharmacokinetic Modelling
Source: Pharmaceutics. 2023 Sep 19;15(9):2348. doi: 10.3390/pharmaceutics15092348 (PMC10536587; doi:10.3390/pharmaceutics15092348)
Supplement: Supplementary file 1 [file pharmaceutics-15-02348-s001.zip › pharmaceutics-2598008-supplementary.pdf]

Supplementary materials

Predicting Volume of Distribution in Neonates: Performance of Physiologically Based Pharmacokinetic Modelling

Pieter-Jan De Sutter *, Phebe Rossignol, Lien Breëns, Elke Gasthuys and An Vermeulen

Department of Bioanalysis, Ghent University, 9000 Ghent, Belgium
* Correspondence: pieterjan.desutter@ugent.be

Supplementary Figure S1

Supplementary Figure S2

Supplementary Figure S3

Supplementary Table S1

Supplementary Table S2

Supplementary Table S3

Supplementary References

Supplementary figures

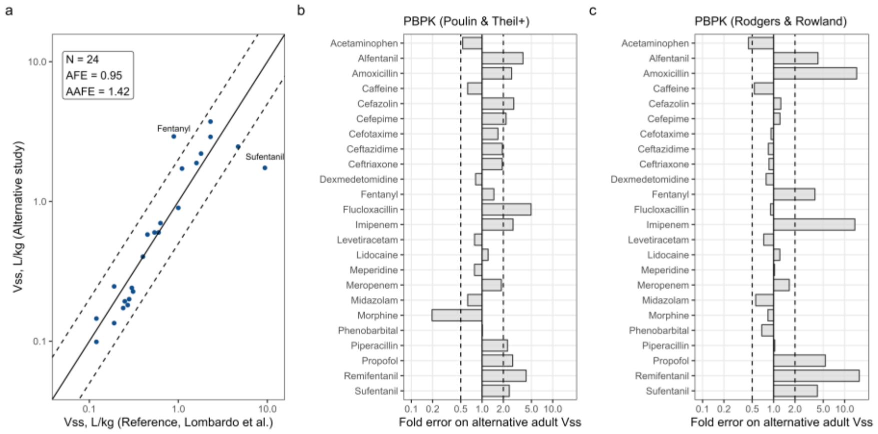

Figure S1. Comparison of alternative volumes of distribution at steady state (Vss) in adults with original reference data (panel **a**) and the accuracy of two physiologically based pharmacokinetic modelling (PBPK) methods in predicting Vss when the alternative Vss is used as comparator (panels **b** and **c**). PBPK predictions are done with either the Poulin & Theil with Berezhkovskiy correction method (panel **b**) or with the Rodgers & Rowland method (panel **c**). Dashed lines denote a twofold interval around the line of unity (solid line). N: number of drugs, AFE: average fold error, AAFE: absolute average fold error.

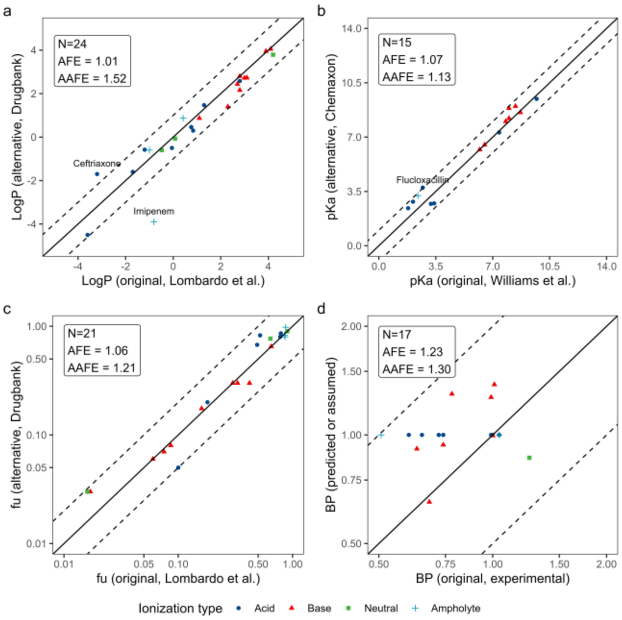

Figure S2. Comparison between original input parameter values (x-axes) and alternative input values (y-axes) of the physiologically based pharmacokinetic (PBPK) models. Input parameters evaluated are LogP (panel **a**), pKa of the strongest acid or base (panel **b**), free fraction in plasma (fu, panel **c**) and the blood-to-plasma ratio (BP, panel **d**). Original values originated from the Lombardo et al. database (1)(LogP and fu), the Williams et al. database (pKa) (2) or a variety of sources (BP). Alternative values were sourced from Drugbank (LogP and fu), predicted with the Chemaxon method (pKa) or predicted using the Simcyp software (BP, only for bases and neutrals, for acids: assumed to be equal to 1). The solid lines denote the line of unity, the dashed lines denote a log unit difference (panels **a** and **b**) or a twofold difference (panels **c** and **d**). N: number of drugs with full data, AFE: average fold error, AAFE: absolute average fold error.

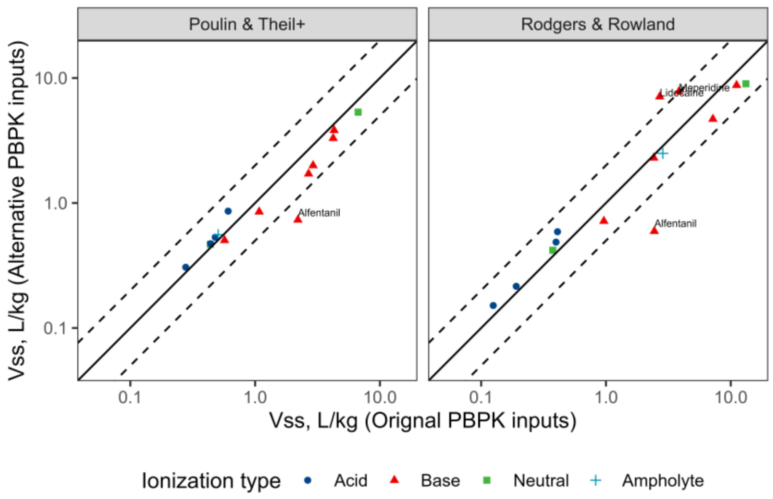

Figure S3. Comparison between physiologically based pharmacokinetic (PBPK) predicted volume of distributions at steady state (Vss) in adults when either the original parameters or alternative input parameters are used. PBPK predictions are done with either the Poulin & Theil with Berezhkovskiy correction method (**left** panel) or with the Rodgers & Rowland method (**right** panel). Input parameter values can be found in Table 1 (original inputs) or supplementary Table 2 (alternative inputs). The solid line denotes the line of unity, while the dashed lines denote a twofold interval around the line of unity.

Supplementary tables

Table S1: Reference- and alternative volumes of distribution at steady state (Vss) in adults

Drug	Reference Vss (L/kg) (1)	Alternative Vss (L/kg)	Source alternative Vss	Fold error (alt/ref)
Acetaminophen	1.00	0.90	(3)	0.90
Alfentanil	0.45	0.58	(4)	1.29
Amoxicillin	0.25	0.19	(5)	0.77
Caffeine	0.63	0.70	(6)	1.11
Cefazolin	0.12	0.10	(7)	0.82
Cefepime	0.28	0.20	(8)	0.71
Cefotaxime	0.19	0.25	(9)	1.30
Ceftazidime	0.31	0.23	(9)	0.73
Ceftriaxone	0.12	0.15	(10)	1.21
Dexmedetomidine	1.60	1.89	(11)	1.18
Fentanyl	0.89	2.92	(12)	<u>3.28</u>
Flucloxacillin	0.19	0.13	(13)	0.71
Imipenem	0.24	0.17	(14)	0.72
Levetiracetam	0.60	0.60	(15)	1.00
Lidocaine	1.80	2.20	(16)	1.22
Meperidine	2.30	3.73	(17)	1.62
Meropenem	0.30	0.24	(18)	0.80
Midazolam	1.10	1.72	(19)	1.56
Morphine	2.30	2.90	(20)	1.26
Phenobarbital	0.54	0.60	(21)	1.11
Piperacillin	0.27	0.18	(22)	0.67
Propofol	4.70	2.47	(23)	0.53
Remifentanil	0.40	0.40	(24)	1.01
Sufentanil	9.40	1.74	(25)	<u>0.19</u>

Table S2: Alternative input values for physiologically based pharmacokinetic (PBPK) models

Drug	LogP (Drugbank)	pka1 (Chemaxon)	fu (Drugbank)	BP (predicted [a] or assumed [b])
Acetaminophen	0.46	9.46	0.825	1.00 [b]
Alfentanil	2.16	6.50	0.080	0.92 [a]
Amoxicillin	0.87	3.23	0.830	1.00[b]
Caffeine	-0.07	neutral	0.770	1.00 [b]
Cefazolin	-0.58	2.84	0.200	1.00 [b]
Cefepime	-4.50	/	0.800	1.00 [b]
Cefotaxime	-0.50	2.73	NA	/
Ceftazidime	-1.60	2.42	0.861	1.00 [b]
Ceftriaxone	-1.70	2.70	0.050	/
Dexmedetomidine	2.80	/	0.060	/
Fentanyl	4.05	8.99	0.175	1.27 [a]
Flucloxacillin	2.58	3.75	NA	/
Imipenem	-3.90	/	0.800	/
Levetiracetam	-0.60	16.09	0.900	/
Lidocaine	2.44	8.01	0.300	1.30 [a]
Meperidine	2.72	8.59	0.300	1.38 [a]
Meropenem	-0.60	/	0.980	1.00 [b]
Midazolam	2.73	6.19	0.030	0.65 [a]
Morphine	0.87	8.21	0.650	1.00 [a]
Phenobarbital	1.47	7.30	0.675	1.00 [b]
Piperacillin	0.30	/	NA	1.00 [b]
Propofol	3.79	neutral	0.030	0.86 [a]
Remifentanil	1.40	/	0.300	/
Sufentanil	3.95	8.86	0.070	0.94 [a]

[a] : predicted by Simcyp V22 from physicochemical data (bases)
[b]: assumed to be equal to 1.00 (acids)
/: not applicable, as original data already used this source
BP: blood to plasma ratio, fu: free fraction in plasma, NA: not available, pKa1: pKa of strongest acid or base (acid in case of ampholyte).

Table S3: Studies describing the volume of distribution at steady state (Vss) in neonates

Drug	Study or sub-study	N	females	Postnatal age (days), range	Gestational age (weeks), range	Body weight (kg), mean	Preterms	Reported Vss (L/kg)
Acetaminophen	van Ganzewinkel et al. (2014)(26)	15	47%	0 - 7	24 - 32	1.21	100%	0.76
Acetaminophen	Cook et al. (2016) (27)	35	50%*	1 - 26	23 - 41	2.06	49%	1.07
Alfentanil	Killian et al. (1990) (28) (PT)	5	50%*	0 - 3	26 - 36	NA	100%	0.84
Alfentanil	Killian et al. (1990) (28) (T)	5	50%*	0 - 3	36 - 38	NA	0%	0.82
Alfentanil	Pokela et al. (1992) (29)	15	50%*	0 - 2	30 - 40	2.50	50%*	0.53
Amoxicillin	Huisman-de Boer et al. (1995) (30)	17	35%	3 - 3	25 - 36	1.18	100%	0.67
Amoxicillin	Charles et al. (1997) (31)	40	50%*	1 - 3	24 - 32	1.12	100%	0.60
Amoxicillin	Pullen et al. (2006) (32)	150	48%	0 - 9	25 - 42	2.29	50%*	0.65
Amoxicillin	Pullen et al. (2007) (33)	32	25%	10 - 52	26 - 41	2.27	50%*	0.66
Amoxicillin	Bijleveld et al. (2018) (34)	125	41%	2 - 5	36 - 42	3.34	0%	0.69
Amoxicillin	Tang et al. (2019) (35)	187	50%*	1 - 37	28 - 41	2.99	50%*	1.21
Caffeine	Charles et al. (2008) (36)	110	53%	1 - 45	24 - 29	0.99	100%	0.85
Cefazolin	Deguchi et al. (1988) (37) (T)	3	33%	5 - 7	39 - 40	3.36	0%	0.23
Cefazolin	Deguchi et al. (1988) (37) (PT)	8	25%	2 - 28	30 - 35	1.85	100%	0.30
Cefazolin	De Cock et al. (2014) (38)	36	50%*	1 - 30	24 - 40	2.76	50%*	0.31
Cefepime	Lima-Rogel et al. (2008) (39)	31	52%	6 - 58	27 - 38	1.40	50%*	0.41
Cefepime	Shoji et al. (2016) (40) (T)	12	50%*	1 - 30	36 - 42	NA	0%	0.35
Cefepime	Shoji et al. (2016) (40) (PT)	32	50%*	1 - 30	22 - 36	NA	100%	0.40
Cefepime	Zhao et al. (2020) (41)	85	50%*	1 - 25	28 - 42	3.21	50%*	0.62
Cefotaxime	Kearns et al. (1989) (42)	18	28%	1 - 7	24 - 32	1.02	100%	0.46
Cefotaxime	Aujard et al. (1989) (43) (PT)	10	50%*	0 - 7	28 - 36	NA	100%	0.38
Cefotaxime	Aujard et al. (1989) (43) (T)	6	50%*	0 - 7	37 - NA	NA	0%	0.45
Cefotaxime	Shang et al. (2022) (44)	51	39%	1 - 3	30 - 41	2.31	12%	0.38
Ceftazidime	Van Den Anker et al. (1995) (45)a	10	50%*	3 - 3	37 - NA	3.06	0%	0.34
Ceftazidime	Van Den Anker et al. (1995) (45)b	9	50%*	3 - 3	37 - NA	3.37	0%	0.34
Ceftazidime	Van Den Anker et al. (1995) (46)	136	50%*	3 - 3	24 - 37	1.41	100%	0.35
Ceftazidime	Van Den Anker et al. (1995) (47)c	13	50%*	3 - 3	26 - 32	1.17	100%	0.32
Ceftazidime	Van Den Anker et al. (1995) (47)d	25	50%*	3 - 3	26 - 32	1.14	100%	0.30
Ceftazidime	Van Den Anker et al. (1995) (48)e	11	50%*	3 - 3	25 - 32	1.15	100%	0.36
Ceftazidime	Van Den Anker et al. (1995) (48)e	11	50%*	10 - 10	25 - 32	1.15	100%	0.29
Ceftazidime	Van Den Anker et al. (1995) (48)f	12	50%*	3 - 3	27 - 32	1.02	100%	0.33
Ceftazidime	Van Den Anker et al. (1995) (48)f	12	50%*	10 - 10	27 - 32	1.02	100%	0.32
Ceftazidime	Wang et al. (2018) (49)	43	50%*	1 - 60	27 - 41	2.73	50%*	0.67
Ceftriaxone	McCracken et al. (1983) (50)	26	50%*	1 - 45	NA - NA	1.83	50%*	0.55
Ceftriaxone	Martin et al. (1984) (51)	12	50%*	1 - 30	NA - NA	2.88	50%*	0.52
Ceftriaxone	Schaad et al. (1985) (52) (T)	6	33%	1 - 7	37 - 41	3.38	0%	0.32
Ceftriaxone	Schaad et al. (1985) (52) (PT)	10	30%	1 - 8	34 - 36	2.36	100%	0.43
Ceftriaxone	Mulhall et al. (1985) (53)g	12	50%*	1 - 16	26 - 41	1.88	50%*	0.33
Ceftriaxone	Mulhall et al. (1985) (53)h	10	50%*	1 - 16	26 - 41	1.88	50%*	0.39
Dexmedetomidine	Chrysostomou et al. (2014) (54)	18	61%	0 - 18	28 - 36	1.70	100%	2.70
Dexmedetomidine	McAdams et al. (2020) (55)	7	50%*	0 - 5	37 - 41	3.51	0%	7.48
Fentanyl	Koehntop et al. (1986) (56)	14	50%*	0 - 14	NA - NA	2.90	50%*	5.10

Fentanyl	Gauntlett et al. (1988) (57)	11	50%*	1 - 59	36 - 40	2.80	0%	8.29
Flucloxacillin	Herngren et al. (1987) (58)	9	50%*	1 - 14	33 - 41	2.49	44%	0.28
Flucloxacillin	Pullen et al. (2006) (59)	55	50%*	0 - 44	26 - 42	1.39	50%*	0.54
Imipenem	Gruber et al. (1985) (60)	10	50%*	1 - 8	NA - NA	2.71	50%*	0.38
Imipenem	Freij et al. (1985) (61)	14	50%*	0 - 10	31 - 42	2.40	50%*	0.66
Imipenem	Reed et al. (1990) (62)	41	51%	1 - 6	22 - 36	1.19	100%	0.55
Imipenem	Yoshizawa et al. (2013) (63)	60	50%	0 - 34	30 - 41	2.93	50%*	0.47
Levetiracetam	Merhar et al. (2011) (64)	18	44%	0 - 32	35 - 41	3.50	50%*	0.89
Levetiracetam	Sharpe et al. (2012) (65)	18	50%	1 - 5	36 - 41	3.24	0%	1.01
Levetiracetam	Lima-Rogel et al. (2018) (66)	20	45%	3 - 25	28 - 40	2.90	30%	0.65
Lidocaine	van den Broek et al. (2011) (67)	46	33%	0 - 10	25 - 43	2.93	39%	2.96
Meperidine	Pokela et al. (1992) (68) (PT+T)	14	36%	0 - 54	32 - 41	3.46	50%*	6.52
Meperidine	Pokela et al. (1992) (68) (PT)	3	33%	1 - 23	28 - 33	1.90	100%	7.17
Meropenem	van den Anker et al. (2009) (69) (PT)	23	43%	0 - 28	29 - 36	1.87	100%	0.52
Meropenem	van den Anker et al. (2009) (69) (T)	15	33%	0 - 28	37 - 42	3.17	0%	0.31
Meropenem	Padari et al. (2012) (70)	19	37%	0 - 56	32 - NA	0.98	50%*	0.31
Meropenem	Lima-Rogel et al. (2021) (71)	40	53%	5 - 28	26 - 41	1.62	50%*	0.46
Midazolam	Jacqz-Aigrain et al. (1990) (72) (T)	7	50%*	2 - 5	37 - 41	3.34	0%	0.90
Midazolam	Jacqz-Aigrain et al. (1990) (72) (PT)	3	50%*	2 - 5	34 - 35	2.57	100%	0.98
Midazolam	Jacqz-Aigrain et al. (1992) (73)	15	50%*	1 - 5	29 - 41	2.30	27%	1.10
Midazolam	Harte et al. (1997) (74)	10	50%*	2 - 4	25 - 30	1.04	100%	1.15
Midazolam	Favié et al. (2019) (75)	118	40%	0 - 5	36 - NA	3.47	0%	1.55
Morphine	Chay et al. (1992) (76) (T)	7	37%	0 - 4	37 - 41	3.22	0%	2.07
Morphine	Chay et al. (1992) (76) (PT)	12	37%	0 - 4	28 - 36	2.19	100%	2.04
Morphine	Pokela et al. (1993) (77) (T)	16	50%*	0 - 57	38 - 42	3.85	0%	1.66
Morphine	Pokela et al. (1993) (77) (PT)	4	50%*	1 - 24	28 - 36	2.08	100%	1.35
Morphine	Farrington et al. (1993) (78) (T)	6	50%*	1 - 6	37 - 40	3.15	0%	6.47
Morphine	Farrington et al. (1993) (78) (PT)	5	50%*	1 - 6	32 - 36	2.34	100%	3.26
Morphine	Anand et al. (2008) (79)	875	50%*	0 - 3	23 - 32	1.04	100%	2.71
Phenobarbital	Heimann et al. (1977) (80)	14	50%*	0 - 28	NA - NA	NA	50%*	0.85
Phenobarbital	Grasela et al. (1985) (81)	59	41%	1 - 16	24 - 42	1.52	78%	0.96
Phenobarbital	Donn et al. (1985) (82)	10	50%*	0 - 6	37 - 41	3.06	0%	0.97
Phenobarbital	Sima et al. (2018) (83)	36	42%	2 - 7	37 - NA	3.19	0%	0.49
Phenobarbital	Favié et al. (2019) (75)	113	37%	2 - 5	36 - NA	3.38	0%	1.03
Phenobarbital	Pokorna et al. (2019) (84)	40	57%	0 - 1	36 - NA	3.26	0%	0.48
Piperacillin	Kacet et al. (1992) (85) (PT)	14	50%*	3 - 3	29 - 35	NA	100%	0.55
Piperacillin	Kacet et al. (1992) (85) (T)	4	50%*	3 - 3	38 - 40	NA	0%	0.54
Piperacillin	Li et al. (2013) (86)	71	50%*	1 - 56	26 - 41	2.76	50%*	0.37
Propofol	Allegaert et al. (2007) (87)	25	16%	1 - 25	26 - 40	2.93	40%	5.56
Propofol	Allegaert et al. (2007) (88) (T)	3	50%*	8 - 25	39 - 40	2.51	0%	3.37
Propofol	Allegaert et al. (2007) (88) (PT)	6	50%*	4 - 25	25 - 36	2.51	100%	4.29
Remifentanil	Ross et al. (2001) (89)	8	50%*	5 - 56	NA - NA	3.74	50%*	0.45
Sufentanil	Greeley et al. (1987) (90)	9	50%*	1 - 30	NA - NA	3.24	50%*	4.15
Sufentanil	Pokorna et al. (2021) (91)	12	75%	0 - 1	37 - 40	3.17	0%	11.8

Sub-study specification: ^{a}: asphyxiated, ^{b}: non-asphyxiated, ^{c}: once daily, ^{d}: twice daily, ^{e}: non exposed to indomethacin, ^{f}: exposed to indomethacin, ^{g}: single dose, ^{h}: multiple dose, (T): term, (PT): preterm.
*: assumed, no data available
N: number of neonates, NA: not available

Supplementary References

1. Lombardo F, Berellini G, Obach RS. Trend Analysis of a Database of Intravenous Pharmacokinetic Parameters in Humans for 1352 Drug Compounds. Drug Metab Dispos. 2018 Nov 1;46(11):1466–77.

2. Williams DA, Lemke TL. pKa values for some drugs and miscellaneous organic acids and bases. In: Foye's Principles of Medicinal Chemistry. 5th ed. Philadelphia: Lippincott, Williams and Wilkins; 2002. p. 1070–9.

3. Forrest JAH, Clements JA, Prescott LF. Clinical Pharmacokinetics of Paracetamol. Clin Pharmacokinet. 1982 Apr 1;7(2):93–107.

4. Persson MP, Nilsson A, Hartvig P. Pharmacokinetics of alfentanil in total i.v. anaesthesia. Br J Anaesth. 1988 Jun;60(7):755–61.

5. Dalhoff A, Koeppe P. Comparative pharmacokinetic analysis of amoxycillin using open two and three-compartment models. Eur J Clin Pharmacol. 1982 May 1;22(3):273–9.

6. Arnaud MJ. The pharmacology of caffeine. In: Jucker E, Meyer U, editors. Progress in Drug Research/Fortschritte der Arzneimittelforschung/Progrès des recherches pharmaceutiques [Internet]. Basel: Birkhäuser; 1987 [cited 2023 May 3]. p. 273–313. (Progress in Drug Research/Fortschritte der Arzneimittelforschung/Progrès des recherches pharmaceutiques). Available from: https://doi.org/10.1007/978-3-0348-9289-6_9

7. Bergan T. Comparative Pharmacokinetics of Cefazolin, Cephalothin, Cephacetril, and Cephapirine after Intravenous Administration. CHE. 1977;23(6):389–404.

8. Pais GM, Chang J, Barreto EF, Stitt G, Downes KJ, Alshaer MH, et al. Clinical Pharmacokinetics and Pharmacodynamics of Cefepime. Clin Pharmacokinet. 2022 Jul;61(7):929–53.

9. Lüthy R, Blaser J, Bonetti A, Simmen H, Wise R, Siegenthaler W. Comparative Multiple-Dose Pharmacokinetics of Cefotaxime, Moxalactam, and Ceftazidime. Reviews of Infectious Diseases. 1982 Nov 1;4(Supplement_3):S581–4.

10. Zhou HH, Chan YP, Arnold K, Sun M. Single-dose pharmacokinetics of ceftriaxone in healthy Chinese adults. Antimicrob Agents Chemother. 1985 Feb;27(2):192–6.

11. Weerink MAS, Struys MMRF, Hannivoort LN, Barends CRM, Absalom AR, Colin P. Clinical Pharmacokinetics and Pharmacodynamics of Dexmedetomidine. Clin Pharmacokinet. 2017;56(8):893–913.

12. Ariano RE, Duke PC, Sitar DS. Population Pharmacokinetics of Fentanyl in Healthy Volunteers. The Journal of Clinical Pharmacology. 2001;41(7):757–63.

13. Landersdorfer CB, Kirkpatrick CMJ, Kinzig-Schippers M, Bulitta JB, Holzgrabe U, Drusano GL, et al. Population Pharmacokinetics at Two Dose Levels and Pharmacodynamic Profiling of Flucloxacillin. Antimicrob Agents Chemother. 2007 Sep;51(9):3290–7.

14. Jaruratanasirikul S, Raungsri N, Punyo J, Sriwiriyajan S. Pharmacokinetics of imipenem in healthy volunteers following administration by 2 h or 0.5 h infusion. Journal of Antimicrobial Chemotherapy. 2005 Dec 1;56(6):1163–5.

15. Patsalos PN. Clinical Pharmacokinetics of Levetiracetam. Clin Pharmacokinet. 2004 Sep 1;43(11):707–24.

16. Bennett PN, Aarons LJ, Bending MR, Steiner JA, Rowland M. Pharmacokinetics of lidocaine and its deethylated metabolite: Dose and time dependency studies in man. Journal of Pharmacokinetics and Biopharmaceutics. 1982 Jun 1;10(3):265–81.

17. Mather LE, Tucker GT, Pflug AE, Lindop MJ, Wilkerson C. Meperidine kinetics in man; Intravenous injection in surgical patients and volunteers. Clinical Pharmacology & Therapeutics. 1975;17(1):21–30.

18. Bax RP, Bastain W, Featherstone A, Wilkinson DM, Hutchison M. The pharmacokinetics of meropenem in volunteers. Journal of Antimicrobial Chemotherapy. 1989 Jan 1;24(suppl_A):311–20.

19. Persson P, Nilsson A, Hartvig P, Tamsen A. Pharmacokinetics of midazolam in total i.v. anaesthesia. Br J Anaesth. 1987 May;59(5):548–56.

20. Hasselström J, Säwe J. Morphine Pharmacokinetics and Metabolism in Humans. Clin Pharmacokinet. 1993 Apr 1;24(4):344–54.

21. Nelson E, Powell JR, Conrad K, Likes K, Byers J, Baker S, et al. Phenobarbital Pharmacokinetics and Bioavailability in Adults. The Journal of Clinical Pharmacology. 1982;22(2–3):141–8.

22. Bulitta JB, Kinzig M, Jakob V, Holzgrabe U, Sörgel F, Holford NHG. Nonlinear pharmacokinetics of piperacillin in healthy volunteers – implications for optimal dosage regimens. Br J Clin Pharmacol. 2010 Nov;70(5):682–93.

23. Eleveld DJ, Proost JH, Cortínez LI, Absalom AR, Struys MMRF. A General Purpose Pharmacokinetic Model for Propofol. Anesthesia & Analgesia. 2014 Jun;118(6):1221–37.

24. Egan TD, Lemmens HJM, Fiset P, Hermann DJ, Muir KT, Stanski DR, et al. The Pharmacokinetics of the New Short-acting Opioid Remifentanil (GI87084B) in Healthy Adult Male Volunteers. Anesthesiology. 1993 Nov 1;79(5):881–92.

25. Bovill JG, Sebel PS, Blackburn CL, Oei-Lim V, Heykants JJ. The Pharmacokinetics of Sufentanil in Surgical Patients. Anesthesiology. 1984 Nov 1;61(5):502–6.

26. van Ganzewinkel C, Derijks L, Anand K, van Lingen R, Neef C, Kramer B, et al. Multiple intravenous doses of paracetamol result in a predictable pharmacokinetic profile in very preterm infants. Acta Paediatrica. 2014;103(6):612–7.

27. Cook SF, Roberts JK, Samiee-Zafarghandy S, Stockmann C, King AD, Deutsch N, et al. Population Pharmacokinetics of Intravenous Paracetamol (Acetaminophen) in Preterm and Term Neonates: Model Development and External Evaluation. Clin Pharmacokinet. 2016 Jan 1;55(1):107–19.

28. Killian A, Davis PJ, Stiller RL, Cicco R, Cook DR, Guthrie RD. Influence of Gestational Age on Pharmacokinetics of Alfentanil in Neonates. DPD. 1990;15:82–5.

29. Pokela ML, Ryhanen PT, Koivisto ME, Olkkola KT, Saukkonen AL. Alfentanil-Induced Rigidity in Newborn Infants. Anesthesia & Analgesia. 1992 Aug;75(2):252.

30. Huisman-de Boer JJ, van den Anker JN, Vogel M, Goessens WH, Schoemaker RC, de Groot R. Amoxicillin pharmacokinetics in preterm infants with gestational ages of less than 32 weeks. Antimicrobial Agents and Chemotherapy. 1995 Feb;39(2):431–4.

31. Charles BG, Preechagoon Y, Lee TC, Steer PA, Flenady VJ, Debuse N. Population Pharmacokinetics of Intravenous Amoxicillin in Very Low Birth Weight Infants. JPharmSci. 1997 Nov 1;86(11):1288–92.

32. Pullen J, Stolk LML, Nieman FHM, Degraeuwe PLJ, van Tiel FH, Zimmermann LJI. Population Pharmacokinetics and Dosing of Amoxicillin in (Pre)term Neonates. Therapeutic Drug Monitoring. 2006 Apr;28(2):226.

33. Pullen J, Driessen M, Stolk LML, Degraeuwe PLJ, van Tiel FH, Neef C, et al. Amoxicillin Pharmacokinetics in (Preterm) Infants Aged 10 to 52 Days: Effect of Postnatal Age. Therapeutic Drug Monitoring. 2007 Jun;29(3):376.

34. Bijleveld Y, Mathôt R, van der Lee J, Groenendaal F, Dijk P, van Heijst A, et al. Population Pharmacokinetics of Amoxicillin in Term Neonates Undergoing Moderate Hypothermia. Clinical Pharmacology & Therapeutics. 2018;103(3):458–67.

35. Tang BH, Wu YE, Kou C, Qi YJ, Qi H, Xu HY, et al. Population Pharmacokinetics and Dosing Optimization of Amoxicillin in Neonates and Young Infants. Antimicrobial Agents and Chemotherapy. 2019 Jan 29;63(2):e02336-18.

36. Charles BG, Townsend SR, Steer PA, Flenady VJ, Gray PH, Shearman A. Caffeine Citrate Treatment for Extremely Premature Infants With Apnea: Population Pharmacokinetics, Absolute Bioavailability, and Implications for Therapeutic Drug Monitoring. Therapeutic Drug Monitoring. 2008 Dec;30(6):709.

37. Deguchi Y, Koshida R, Nakashima E, Watanabe R, Taniguchi N, Ichimura F, et al. Interindividual changes in volume of distribution of cefazolin in newborn infants and its prediction based on physiological pharmacokinetic concepts. JPharmSci. 1988 Aug 1;77(8):674–8.

38. De Cock RFW, Smits A, Allegaert K, de Hoon J, Saegeman V, Danhof M, et al. Population pharmacokinetic modelling of total and unbound cefazolin plasma concentrations as a guide for dosing in preterm and term neonates. Journal of Antimicrobial Chemotherapy. 2014 May 1;69(5):1330–8.

39. Lima-Rogel V, Medina-Rojas EL, Del Carmen Milán-Segovia R, Noyola DE, Nieto-Aguirre K, López-Delarosa A, et al. Population pharmacokinetics of cefepime in neonates with severe nosocomial infections. J Clin Pharm Ther. 2008 Jun;33(3):295–306.

40. Shoji K, Bradley JS, Reed MD, van den Anker JN, Domonoske C, Capparelli EV. Population Pharmacokinetic Assessment and Pharmacodynamic Implications of Pediatric Cefepime Dosing for Susceptible-Dose-Dependent Organisms. Antimicrob Agents Chemother. 2016 Apr;60(4):2150–6.

41. Zhao Y, Yao BF, Kou C, Xu HY, Tang BH, Wu YE, et al. Developmental Population Pharmacokinetics and Dosing Optimization of Cefepime in Neonates and Young Infants. Frontiers in Pharmacology [Internet]. 2020 [cited 2023 Apr 27];11. Available from: https://www.frontiersin.org/articles/10.3389/fphar.2020.00014

42. Kearns GL, Jacobs RF, Thomas BR, Darville TL, Trang JM. Cefotaxime and desacetylcefotaxime pharmacokinetics in very low birth weight neonates. The Journal of Pediatrics. 1989 Mar 1;114(3):461–7.

43. Aujard Y, Brion F, Jacqz-Aigrain E, Kasse MC, Chretien P, Criqui C, et al. Pharmacokinetics of cefotaxime and desacetylcefotaxime in the newborn. Diagnostic Microbiology and Infectious Disease. 1989 Jan 1;12(1):87–91.

44. Shang ZH, Wu YE, Lv DM, Zhang W, Liu WQ, van den Anker J, et al. Optimal dose of cefotaxime in neonates with early-onset sepsis: A developmental pharmacokinetic model-based evaluation. Frontiers in Pharmacology [Internet]. 2022 [cited 2023 Apr 27];13. Available from: https://www.frontiersin.org/articles/10.3389/fphar.2022.916253

45. Van Den Anker JN, Van Der Heijden BJ, Hop WCJ, Schoemaker RC, Broerse HM, Neijens HJ, et al. The Effect of Asphyxia on the Pharmacokinetics of Ceftazidime in the Term Newborn. Pediatr Res. 1995 Nov;38(5):808–11.

46. van den Anker JN, Schoemaker RC, Hop WCJ, van der Heijden BJ, Weber A, Sauer PJJ, et al. Ceftazidime pharmacokinetics in preterm infants: Effects of renal function and gestational age. Clinical Pharmacology & Therapeutics. 1995;58(6):650–9.

47. van den Anker JN, Schoemaker RC, van der Heijden BJ, Broerse HM, Neijens HJ, de Groot R. Once-daily versus twice-daily administration of ceftazidime in the preterm infant. Antimicrobial Agents and Chemotherapy. 1995 Sep;39(9):2048–50.

48. van den Anker JN, Hop WC, Schoemaker RC, van der Heijden BJ, Neijens HJ, de Groot R. Ceftazidime pharmacokinetics in preterm infants: effect of postnatal age and postnatal exposure to indomethacin. Br J Clin Pharmacol. 1995 Nov;40(5):439–43.

49. Wang H, Li X, Sun S, Mao G, Xiao P, Fu C, et al. Population Pharmacokinetics and Dosing Simulations of Ceftazidime in Chinese Neonates. JPharmSci. 2018 May 1;107(5):1416–22.

50. McCracken GH, Siegel JD, Threlkeld N, Thomas M. Ceftriaxone pharmacokinetics in newborn infants. Antimicrobial Agents and Chemotherapy. 1983 Feb;23(2):341–3.

51. Martin E, Koup JR, Paravicini U, Stoeckel K. Pharmacokinetics of ceftriaxone in neonates and infants with meningitis. The Journal of Pediatrics. 1984 Sep 1;105(3):475–81.

52. Schaad UB, Hayton WL, Stoeckel K. Single-dose ceftriaxone kinetics in the newborn. Clinical Pharmacology & Therapeutics. 1985;37(5):522–8.

53. Mulhall A, de Louvois J, James J. Pharmacokinetics and safety of ceftriaxone in the neonate. Eur J Pediatr. 1985 Nov 1;144(4):379–82.

54. Chrysostomou C, Schulman SR, Herrera Castellanos M, Cofer BE, Mitra S, da Rocha MG, et al. A Phase II/III, Multicenter, Safety, Efficacy, and Pharmacokinetic Study of Dexmedetomidine in Preterm and Term Neonates. The Journal of Pediatrics. 2014 Feb 1;164(2):276-282.e3.

55. McAdams RM, Pak D, Lalovic B, Phillips B, Shen DD. Dexmedetomidine Pharmacokinetics in Neonates with Hypoxic-Ischemic Encephalopathy Receiving Hypothermia. Anesthesiology Research and Practice. 2020 Feb 25;2020:e2582965.

56. Koehntop DE, Rodman JH, Brundage DM, Hegland MG, Buckley JJ. Pharmacokinetics of fentanyl in neonates. Anesth Analg. 1986 Mar;65(3):227–32.

57. Gauntlett IS, Fisher DM, Hertzka RE, Kuhis E, Spellman MJ, Rudolph C. Pharmacokinetics of Fentanyl in Neonatal Humans and Lambs: Effects of Age. Anesthesiology. 1988 Nov 1;69(5):683–7.

58. Herngren L, Ehrnebo M, Broberger U. Pharmacokinetics of free and total flucloxacilin in newborn infants. Eur J Clin Pharmacol. 1987 Jul 1;32(4):403–9.

59. Pullen J, de Rozario L, Stolk LML, Degraeuwe PLJ, van Tiel FH, Zimmermann LJI. Population Pharmacokinetics and Dosing of Flucloxacillin in Preterm and Term Neonates. Therapeutic Drug Monitoring. 2006 Jun;28(3):351.

60. Gruber WC, Rench MA, Garcia-Prats JA, Edwards MS, Baker CJ. Single-dose pharmacokinetics of imipenem-cilastatin in neonates. Antimicrobial Agents and Chemotherapy. 1985 Apr;27(4):511–4.

61. Freij BJ, McCracken GH, Olsen KD, Threlkeld N. Pharmacokinetics of imipenem-cilastatin in neonates. Antimicrobial Agents and Chemotherapy. 1985 Apr;27(4):431–5.

62. Reed MD, Kliegman RM, Yamashita TS, Myers CM, Blumer JL. Clinical pharmacology of imipenem and cilastatin in premature infants during the first week of life. Antimicrobial Agents and Chemotherapy. 1990 Jun;34(6):1172–7.

63. Yoshizawa K, Ikawa K, Ikeda K, Ohge H, Morikawa N. Population Pharmacokinetic–Pharmacodynamic Target Attainment Analysis of Imipenem Plasma and Urine Data in Neonates and Children. The Pediatric Infectious Disease Journal. 2013 Nov;32(11):1208.

64. Merhar SL, Schibler KR, Sherwin CM, Meinzen-Derr J, Shi J, Balmakund T, et al. Pharmacokinetics of Levetiracetam in Neonates with Seizures. The Journal of Pediatrics. 2011 Jul 1;159(1):152-154.e3.

65. Sharpe CM, Capparelli EV, Mower A, Farrell MJ, Soldin SJ, Haas RH. A seven-day study of the pharmacokinetics of intravenous levetiracetam in neonates: marked changes in pharmacokinetics occur during the first week of life. Pediatr Res. 2012 Jul;72(1):43–9.

66. Lima-Rogel V, López-López EJ, Medellín-Garibay SE, Gómez-Ruiz LM, Romero-Méndez C, Milán-Segovia RC, et al. Population pharmacokinetics of levetiracetam in neonates with seizures. Journal of Clinical Pharmacy and Therapeutics. 2018;43(3):422–9.

67. van den Broek MPH, Huitema ADR, van Hasselt JGC, Groenendaal F, Toet MC, Egberts TCG, et al. Lidocaine (Lignocaine) Dosing Regimen Based upon a Population Pharmacokinetic Model for Preterm and Term Neonates with Seizures. Clin Pharmacokinet. 2011 Jul 1;50(7):461–9.

68. Pokela ML, Olkkola KT, Koivisto M, Ryhänen P. Pharmacokinetics and pharmacodynamics of intravenous meperidine in neonates and infants. Clinical Pharmacology & Therapeutics. 1992;52(4):342–9.

69. van den Anker JN, Pokorna P, Kinzig-Schippers M, Martinkova J, de Groot R, Drusano GL, et al. Meropenem Pharmacokinetics in the Newborn. Antimicrobial Agents and Chemotherapy. 2009 Sep;53(9):3871–9.

70. Padari H, Metsvaht T, Kõrgvee LT, Germovsek E, Ilmoja ML, Kipper K, et al. Short versus Long Infusion of Meropenem in Very-Low-Birth-Weight Neonates. Antimicrobial Agents and Chemotherapy. 2012 Sep;56(9):4760–4.

71. Lima-Rogel V, Olguín-Mexquitic L, Kühn-Córdova I, Correa-López T, Romano-Aguilar M, Romero-Méndez M del C, et al. Optimizing Meropenem Therapy for Severe Nosocomial Infections in Neonates. JPharmSci. 2021 Oct 1;110(10):3520–6.

72. Jacqz-Aigrain E, Wood C, Robieux I. Pharmacokinetics of midazolam in critically ill neonates. Eur J Clin Pharmacol. 1990 Aug 1;39(2):191–2.

73. Jacqz-Aigrain E, Daoud P, Burtin P, Maherzi S, Beaufils F. Pharmacokinetics of midazolam during continuous infusion in critically ill neonates. Eur J Clin Pharmacol. 1992 Mar 1;42(3):329–32.

74. Harte G, Gray P, Lee T, Steer P, Charles B. Haemodynamic responses and population pharmacokinetics of midazolam following administration to ventilated, preterm neonates. Journal of Paediatrics and Child Health. 1997;33(4):335–8.

75. Favié LMA, Groenendaal F, van den Broek MPH, Rademaker CMA, de Haan TR, van Straaten HLM, et al. Phenobarbital, Midazolam Pharmacokinetics, Effectiveness, and Drug-Drug Interaction in Asphyxiated Neonates Undergoing Therapeutic Hypothermia. Neonatology. 2019;116(2):154–62.

76. Chay PCW, Duffy BJ, Walker JS. Pharmacokinetic-pharmacodynamic relationships of morphine in neonates. Clinical Pharmacology & Therapeutics. 1992;51(3):334–42.

77. Pokela ML, Olkkola KT, Seppälä T, Koivisto M. Age-Related Morphine Kinetics in Infants. DPD. 1993;20:26–34.

78. Farrington EA, McGuinness GA, Johnson GF, Erenberg A, Leff RD. Continuous Intravenous Morphine Infusion in Postoperative Newborn Infants. Am J Perinatol. 1993 Jan;10(1):84–7.

79. Anand KJS, Anderson BJ, Holford NHG, Hall RW, Young T, Shephard B, et al. Morphine pharmacokinetics and pharmacodynamics in preterm and term neonates: secondary results from the NEOPAIN trial. British Journal of Anaesthesia. 2008 Nov 1;101(5):680–9.

80. Heimann G, Gladtke E. Pharmacokinetics of phenobarbital in childhood. Eur J Clin Pharmacol. 1977 Jul 1;12(4):305–10.

81. Grasela TH, Donn SM. Neonatal population pharmacokinetics of phenobarbital derived from routine clinical data. Dev Pharmacol Ther. 1985;8(6):374–83.

82. Donn SM, Grasela TH, Goldstein GW. Safety of a higher loading dose of phenobarbital in the term newborn. Pediatrics. 1985 Jun;75(6):1061–4.

83. Šíma M, Pokorná P, Hartinger J, Slanař O. Estimation of initial phenobarbital dosing in term neonates with moderate-to-severe hypoxic ischaemic encephalopathy following perinatal asphyxia. J Clin Pharm Ther. 2018 Apr;43(2):196–201.

84. Pokorná P, Posch L, Šíma M, Klement P, Slanar O, van den Anker J, et al. Severity of asphyxia is a covariate of phenobarbital clearance in newborns undergoing hypothermia. J Matern Fetal Neonatal Med. 2019 Jul;32(14):2302–9.

85. Kacet N, Roussel-Delnallez M, Gremillet C, Dubos JP, Storme L, Lequien P. Pharmacokinetic study of piperacillin in newborns relating to gestational and postnatal age. The Pediatric Infectious Disease Journal. 1992 May;11(5):365.

86. Li Z, Chen Y, Li Q, Cao D, Shi W, Cao Y, et al. Population pharmacokinetics of piperacillin/tazobactam in neonates and young infants. Eur J Clin Pharmacol. 2013 Jun 1;69(6):1223–33.

87. Allegaert K, Peeters MY, Verbesselt R, Tibboel D, Naulaers G, de Hoon JN, et al. Inter-individual variability in propofol pharmacokinetics in preterm and term neonates. British Journal of Anaesthesia. 2007 Dec 1;99(6):864–70.

88. Allegaert K, Hoon JD, Verbesselt R, Naulaers G, Murat I. Maturational pharmacokinetics of single intravenous bolus of propofol. Pediatric Anesthesia. 2007;17(11):1028–34.

89. Ross AK, Davis PJ, Dear G deL, Ginsberg B, McGowan FX, Stiller RD, et al. Pharmacokinetics of Remifentanil in Anesthetized Pediatric Patients Undergoing Elective Surgery or Diagnostic Procedures. Anesthesia & Analgesia. 2001 Dec;93(6):1393.

90. Greeley WJ, de Bruijn NP, Davis DP. Sufentanil Pharmacokinetics in Pediatric Cardiovascular Patients. Anesthesia & Analgesia. 1987 Nov;66(11):1067.

91. Pokorná P, Šíma M, Koch B, Tibboel D, Slanař O. Sufentanil Disposition and Pharmacokinetic Model-Based Dosage Regimen for Sufentanil in Ventilated Full-Term Neonates. PHA. 2021;106(7–8):384–9.
